# An audit of antibiotic dosing according to renal function or renal replacement therapy in critical care

**DOI:** 10.1186/cc9638

**Published:** 2011-03-11

**Authors:** KD Donnelly, KD Smith, JJ Coleman, D Westwood, AN Billington

**Affiliations:** 1 UHB Trust, Birmingham, UK

## Introduction

An audit of antibiotic dosing was conducted over the four critical care units at University Hospital Birmingham. The prescribed dose of four antibiotics (co-amoxiclav, meropenem, tazocin and ciprofloxacin) was audited against local prescribing guidance based on renal function and use of renal replacement therapy (RRT).

## Methods

The electronic prescribing system was interrogated for all prescriptions of the intravenous (i.v.) form of the antibiotics during 2009. Antibiotic, dose and frequency, estimated glomerular filtration rate (eGFR), prescriptions of diasylate solution (indicating RRT), and number of administrations were recorded. One-off prescriptions and those without recent eGFR were discarded. A total of 2,472 records were included. Prescriptions were grouped by unit and antibiotic, then by renal function (normal, mild, moderate or severe impairment) or RRT, and by one of four categories (appropriate, underdosing, overdosing or incorrect regimen).

## Results

Of the 2,472 prescriptions, 2,004 (81.1%) were correctly prescribed with regards to renal function and RRT. The total numbers of prescriptions per antibiotic are as follows: coamoxiclav (631 prescriptions, of these 94.9% correct), ciprofloxacin (282, 98.9%), tazocin (696, 80.6%) and meropenem (863, 65.6%). On Unit 3, tazocin was underdosed in cases of normal renal function (15.2% of their ward's prescriptions with regard to that antibiotic; median administrations 3, range 1 to 15), and during RRT (6.5%; 6, 0 to 29). On Unit 4, tazocin was underdosed during mild renal failure (7.0%; 9, 2 to 81) and during RRT (7.7%; 10, 3 to 30). Meropenem was overdosed during RRT on Unit 1 (6.1%; 4, 0 to 30), Unit 3 (20%, 15, 1 to 38) and Unit 4 (13%; 15, 1 to 31), and underdosed during RRT on Unit 4 (17.9%; 7.5, 1 to 28).

## Conclusions

Tazocin was frequently underdosed in this critically ill population. It is possible that the minimum inhibitory concentration was not reached in some patients, with the associated risk of treatment failure [1,2]. Meropenem was underdosed on one unit; however, overdosing was more common. The clinical significance of this is equivocal as raised peak levels can be advantageous [3]. The electronic prescribing system currently lacks renal dosing decision support; this audit suggests a potential benefit to the integration of antibiotic prescribing guidelines.
